# Characterization of Primary Cilia Formation in Human ESC-Derived Retinal Organoids

**DOI:** 10.1155/2023/6494486

**Published:** 2023-01-13

**Authors:** Ke Ning, Ziming Luo, Tia J. Kowal, Matthew Tran, Rishab Majumder, Trent M. Jarin, Albert Y. Wu, Jeffrey L. Goldberg, Yang Sun

**Affiliations:** ^1^Department of Ophthalmology, Stanford University School of Medicine, Palo Alto, CA, USA; ^2^Palo Alto Veterans Administration, Palo Alto, CA, USA

## Abstract

**Objectives:**

Primary cilia are conserved organelles found in polarized mammalian cells that regulate neuronal growth, migration, and differentiation. Proper cilia formation is essential during eye development. Our previous reports found that both amacrine and retinal ganglion cells (RGCs) contain primary cilia in primate and rodent retinas. However, whether primary cilia are present in the inner retina of human retinal organoids remains unknown. The purpose of this study is to characterize the primary cilia distribution in human embryonic stem cell (hESC-derived retinal organoid development.

**Materials and Methods:**

Retinal organoids were differentiated from a hESC line, harvested at various developmental timepoints (day 44-day 266), and immunostained with antibodies for primary cilia, including Arl13b (for the axoneme), AC3, and Centrin3 (for the basal body). AP2*α*, Prox1, GAD67, Calretinin, GFAP, PKC*α*, and Chx10 antibodies as well as Brn3b-promoted tdTomato expression were used to visualize retinal cell types.

**Results:**

A group of ciliated cells were present in the inner aspects of retinal organoids from day 44 to day 266 in culture. Ciliated Chx10-positive retinal progenitor cells, GFAP-positive astrocytes, and PKC*α*-positive rod-bipolar cells were detected later during development (day 176 to day 266). Ciliation persisted during all stages of retinal developmental in AP2*α*-positive amacrine cells, but it was decreased in Brn3b-positive retinal ganglion cells (RGCs) at later time points. Additionally, AC3-positive astrocytes significantly decreased during the later stages of organoid formation.

**Conclusions:**

Amacrine cells in retinal organoids retain cilia throughout development, whereas RGC ciliation gradually and progressively decreases with organoid maturation.

## 1. Introduction

Primary cilia are microtubule-based cellular organelles located at the apical surface of most mammalian cells [1–4]. Many transmembrane proteins localized within cilia play critical roles in transducing external signals into cells to control cellular processes such as cell proliferation, differentiation, and maturation [4–8]. Several cilia-regulated signaling pathways have been well described, including sonic hedgehog and Wnt signaling [9–14]. Mutations in genes that influence either the structure or function of primary cilia result in a group of multiorgan disorders called ciliopathies, which predominantly affect the eye, kidney, and brain [15–20]. In the central nervous system, primary cilia play crucial roles in tissue patterning, neurogenesis, neuronal maturation, and survival [21–24]. In the retina, primary cilia participate directly in the survival and proliferation of retinal progenitor cells [21, 25]. It is also well known that eye patterning and morphogenesis of mouse eyes are dependent upon proper ciliogenesis [26–30]. Most of our knowledge comes from investigations of cilia within photoreceptors, and much less is known about the role of cilia in nonphotoreceptors in the retina [6, 28, 31].

In the brain and retina, ADP-ribosylation factor-like 13B (Arl13b) and type 3 adenylyl cyclase (AC3) are commonly used markers for identifying primary cilia [32–34]. Arl13b belongs to the ARF/ARL family of small GTPases and is highly enriched in primary cilium membranes [35, 36]. Mutations of Arl13b were identified as causative for Joubert syndrome, a form of ciliopathy characterized by cerebellar and brainstem defects as well as retinal dystrophy [19, 37–41]. In rodents, Arl13b plays a key role in the development of the outer segment of photoreceptors and eye formation; loss of Arl13b results in premature photoreceptor degeneration [36]. AC3, another neuronal marker of cilia, regulates cyclic AMP (cAMP) levels in cilia and downstream signaling cascades [42, 43]. Previous studies demonstrated cAMP signaling to be crucial for neuronal survival and axonal growth. The loss of AC3 in olfactory neurons disrupts glomeruli formation and proper olfactory axon projection in mice [44]. Recently, we have also demonstrated that most retinal ganglion cells (RGC) have AC3-positive primary cilia in adult mice, suggesting that AC3 may play a key role in the postnatal development and maintenance of RGC homeostasis [45].

Stem cell-derived retinal organoids have become a model to study mammalian eye development. The retinal organoids, which are derived from human embryonic stem cells (hESCs) or induced pluripotent stem cells (iPSCs), mimic different aspects of the native human retina, offering an ex vivo model system for studying the mechanisms underlying neurodevelopment and diseases [46–53]. A major limitation of retinal organoids is the gradual degeneration and loss of RGCs in long-term organoid cultures [48–50, 52]. Since primary cilia are essential for neural development and differentiation, studies of primary cilia and their roles in retinal organoids may provide unique insights into their functions in these processes. However, the expression and pattern of expression of primary cilia in nonphotoreceptors in retinal organoids are unknown.

This study is aimed at determining the spatial and temporal distribution of primary cilia in hESC-derived retinal organoids, with a particular focus on amacrine cells and RGCs. Analysis using immunofluorescence techniques revealed stable expression of Arl13b-positive primary cilia in amacrine cells during late stages of retinal organoid development along with significant decrease in ciliation of RGCs. Additionally, there were significantly fewer RGCs with AC3-positive primary cilia in these organoids than in human adult retina, which suggests a role for AC3 in RGC maturation and survival.

## 2. Materials and Methods

### 2.1. Reagents

Detailed description for antibodies and their working concentrations are as follows: anti-Centrin3 mouse antibody (IF: 1 : 500) was obtained from Abnova (H00001070-M01); anti-Chx10 chicken antibody (IF: 1 : 500) was obtained from ThermoFisher Scientific (PA1-12565); anti-AC3 rabbit antibody (IF: 1 : 2000), anti-PCK*α* antibody (IF: 1 : 1000), and anti-GFAP chicken antibody (IF: 1 : 2000) were obtained from Abcam (ab125093; ab19031; ab4674); anti-calretinin rabbit antibody (IF: 1 : 500), anti-gamma-tubulin rabbit antibody (IF: 1 : 1000), anti-IFT88 rabbit antibody (IF:1 : 500), and anti-Arl13b rabbit antibody (IF: 1 : 2000) were obtained from Proteintech (12278-1-AP, 66320-1, 13967-1-AP, 17711-1-AP); anti-GAD67 mouse antibody (IF: 1 : 500) and anti-Prox1 rabbit antibody (IF: 1 : 500) were obtained from Millipore Sigma (AB5475); anti-Arl13b mouse antibody (IF: 1 : 2000) was obtained from Antibodies Inc. (N295B/66); anti-RBPMS guinea-pig antibody (IF: 1 : 2000) was obtained from Phosphosolutions (1832); anti-Osteopontin mouse antibody (IF: 1 : 2000) was obtained from DSHB (MPIIIB10); and anti-cleaved Caspase-3 antibody (IF: 1 : 400) was obtained from Cell Signaling #9661. The primary antibodies were detected using Alexa Fluor 488, 647, and 594 IgG secondary antibodies (IF: 1 : 200) purchased from Life Technologies. ProLong Gold Antifade Mount and DAPI were obtained from Introvitrogen.

### 2.2. Retinal Organoid Culturing

The Brn3b-Tdtomato hESC line was a gift from Donald Zack lab and cultured in the Byers Eye Institute Laboratory [53, 54]. The hESC were cultured in a feeder-free system in StemFlex medium (No. A3349401; Thermo Fisher Scientific, Waltham, MA, USA) at 37°C in the presence of 5% CO2. The cells were differentiated into retinal organoids following previously reported protocols [53]. Briefly, hES cells were induced to form embryoid bodies and sequentially differentiated in the medium supplemented with N2 (No. 17,502–048; Thermo Fisher Scientific) and B27 (No. 17,504,044; Thermo Fisher Scientific). Day 0 was the day on which embryonic bodies (EBs) form, and Day 28 was the day on which retinal organoids were cultured in suspension (differentiation week 4 or Dwk 4). In addition, the hESCs used to derive the retinal organoids were modified such that Brn3b, a marker of mature retinal ganglion cells, was conjugated to the fluorophore tdTomato. In this way, upon maturation the retinal ganglion cells were easily identifiable through detection of tdTomato. As expected, the photoreceptors were observed on the outer region of the organoids and the Brn3b-tdTomato positive RGCs were observed on the opposite, inner side of the organoids.

### 2.3. Human Donor Retina and Tissue Preparation

Under the approval of Stanford University's Institutional Review Board and in compliance with the Declaration of Helsinki, human eyes were obtained from donors through the National Disease Resource Interchange (NDRI). Tissue was collected from a 68-year-old donor with no reported history of eye disease. During the procedure, the retina was carefully removed around the edge of the optic disc and fixed in a fresh paraformaldehyde solution (4% PFA; Sigma, St. Louis, MO) in phosphate-buffered saline (1XPBS) within 10 hours of death. After removing the retina, it was placed in methanol for 30 minutes, and then in 1xPBS for 5 minutes. Whole mount immunolabeling was conducted on 0.5 × 0.5 cm retina chunks obtained from the peripheral retina.

### 2.4. Immunofluorescence Staining

The retinal organoids were fixed in 4% PFA for 30 minutes and mounted in O.C.T. Tissue-Tek (Sakura Finetek) on dry ice, followed by cryostat sectioning at 10 *μ*m thickness (CM1860, Leica Biosystems, Wetzlar, Germany). The mounted sections or fixed human retina chunks were washed three times with PBS and incubated at room temperature for two hours in blocking buffer containing 10% goat serum and 0.3% Triton X-100. The primary antibodies were incubated overnight at 4°C with organoid sections or human retina after blocking. The sections or tissues were then washed three times with PBS for 15 minutes, and then incubated for one hour at room temperature with the blocking buffer containing secondary antibodies. DAPI was used to stain the nuclei. After three washes with PBS, the slides or tissues were mounted with ProLong Gold (Life Technologies) on cover slips. An LSM880 Zeiss confocal microscope was used for capturing images.

### 2.5. Quantification

**Figure 1:**
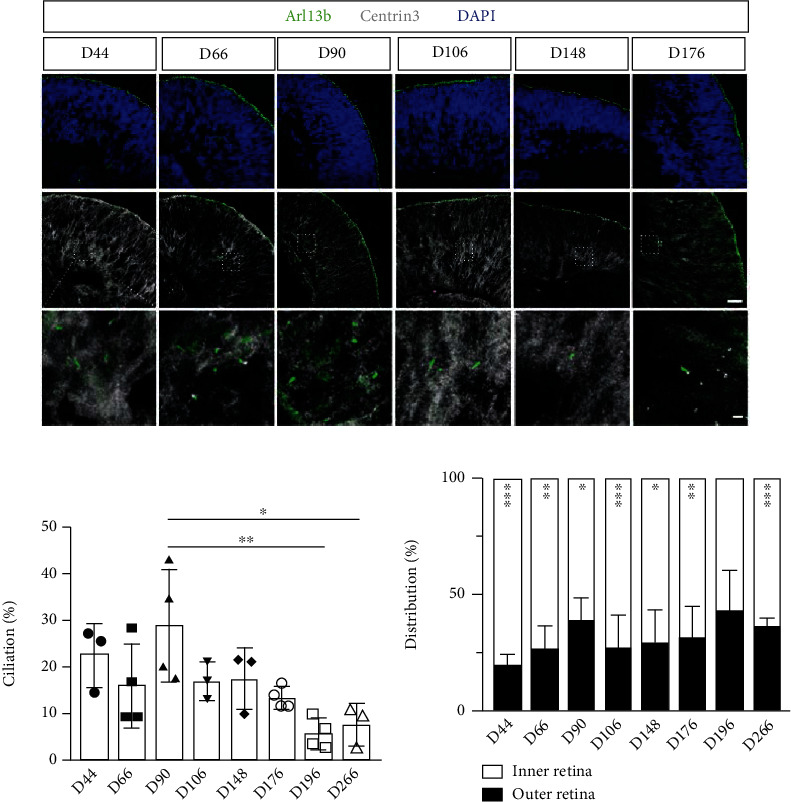
Primary cilia are expressed in the inner part of hESC-derived retinal organoids and their numbers decrease in later developmental stages. (a) Confocal images of retinal organoid sections from different developmental stages immunostained with two primary ciliary markers, Arl13b (green) and Centrin3 (gray) to reveal ciliary axoneme and basal body, respectively, (scale bar: 10 *μ*m; magnified, 1 *μ*m). Nuclei are visualized by DAPI (blue). (b) Quantitative analyses reveals that the ciliation (the number of cells expressing primary cilia) of retinal organoids is at its peak at day 90 and significantly declines at day 196 and day 266 (^∗∗^*P* = 0.003 and ^∗^*P* = 0.014, One-way ANOVA). (c) Quantification of the percentage of ciliated retinal cells distributed in the outer and inner sides of retinal organoids. (Day 44: *P* < 0.001; day 66: *P* = 0.004; day 90: *P* = 0.025; day 106: *P* = 0.003; day 148: *P* = 0.020; day 176: *P* = 0.006; day 196: *P* = 0.314; and day 266: *P* = 0.001; unpaired *t*-test).

**Figure 2:**
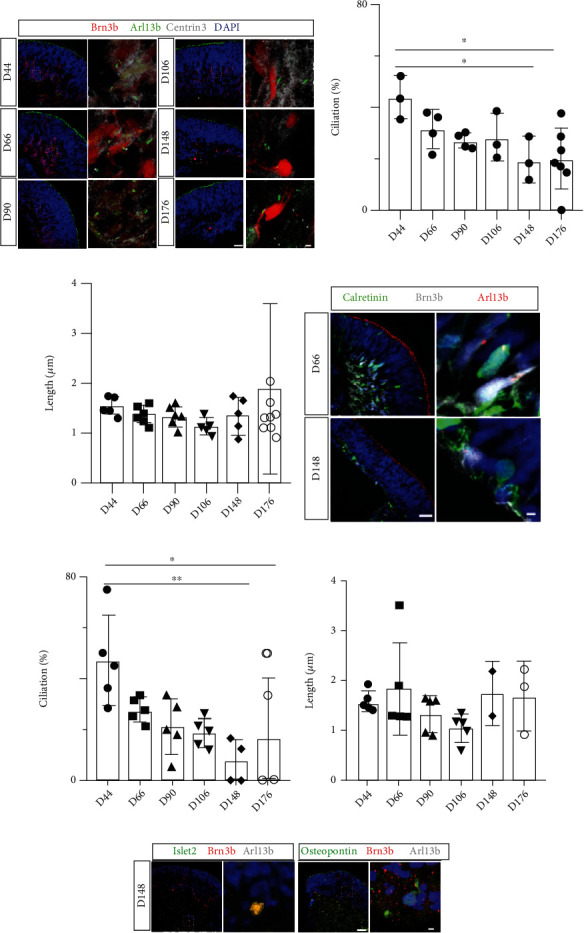
Brn3b-positive RGCs lose primary cilia during later developmental stages of retinal development. (a) Confocal images of retinal organoids show RGCs visualized by Brn3b (red); primary cilia are identified by costaining for Arl13b (green) and Centrin3 (gray), markers for the ciliary axoneme and basal body, respectively. (b) Quantitative assessment confirms that ciliation at days 148 and 176 of retinal organoid development is significantly reduced compared to day 44. (c) Significant variations in ciliary length are not observed. (d) Calretinin-positive RGCs are readily identified by calretinin (green) coexpression with markers for RGCs (Brn3b, gray) and cilia (Arl13b, red). (e) At days 148 and 176 of retinal organoid development, ciliation is significantly decreased compared to day 44. (f) No significant changes in ciliary length are observed in calretinin-positive RGCs during retinal organoid development. Nuclei are stained with DAPI (blue). Scale bar: 10 *μ*m; magnified, 1 *μ*m. (g) Confocal images of day 148 retinal organoids show RGCs visualized by Brn3b (red), RGC subtype markers by Islet2 or Osteopontin (green), and primary cilia by Arl13b (gray).

**Figure 3:**
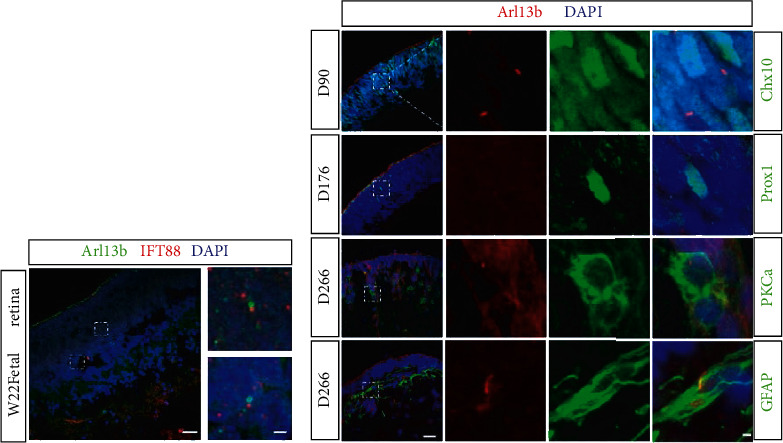
Primary cilia are present in Chx10-positive, GFAP-positive, and PKC*α*-positive retinal cells. (a) Photomicrograph of primary cilia in sections from human fetal retina (week 22, *n* = 1). Arl13b (in green) and IFT88 (in red) antibodies were used as cilia markers. (b) Representative confocal images of retinal organoid sections stained with cell and primary cilia markers (Arl13b in red). Chx10: marker for retinal progenitor cells; Prox1: marker for horizontal cells: PKC*α*: marker for rod-bipolar cells; and GFAP: marker for astrocytes. All cell markers are labeled in green. Nuclei are stained by DAPI (blue). Scale bar: 10 *μ*m; magnified, 1 *μ*m.

**Figure 4:**
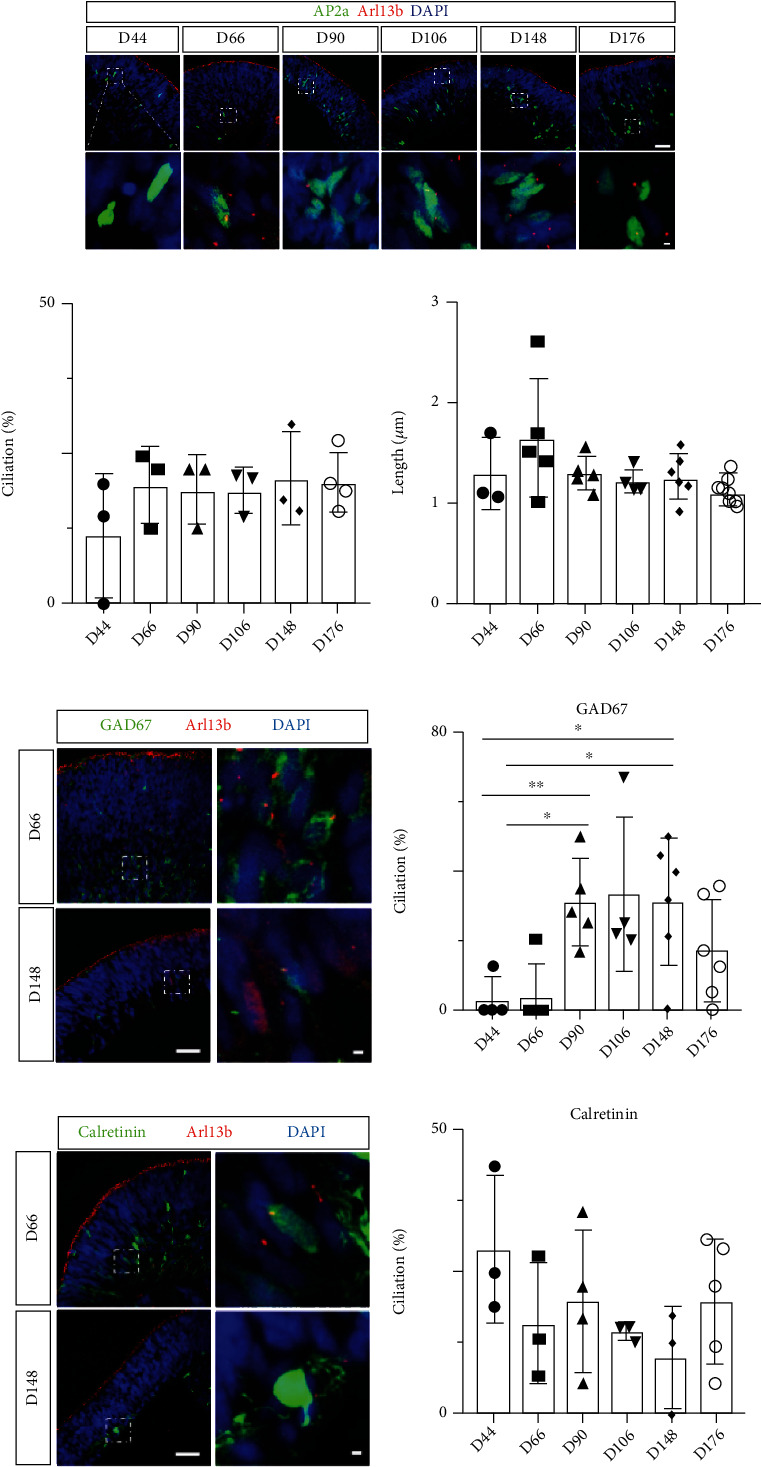
AP2*α*-positive amacrine cells stably express primary cilia in hESC-derived retinal organoids. (a) Confocal images of retinal organoid sections (from day 44 to day 176) costained with AP2*α* (in green) and Arl13b (in red). (b, c) Quantification of ciliation and ciliary length of AP2*α*-positive amacrine cells during retinal organoid development. (d) Representative images of retinal organoid sections costained with GAD67 (green), AP2*α* (in gray, not shown), and Arl13b (red). (e) Representative confocal images of retinal organoids immunostained for calretinin (green), AP2*α* (in gray, not shown), and Arl13b (red). (f, g) Quantification of ciliation of GAD67-positive (f) and calretinin-positive (g) amacrine cells. Nuclei are visualized by staining with DAPI (blue). Scale bar: 10 *μ*m; magnified, 1 *μ*m.

**Figure 5:**
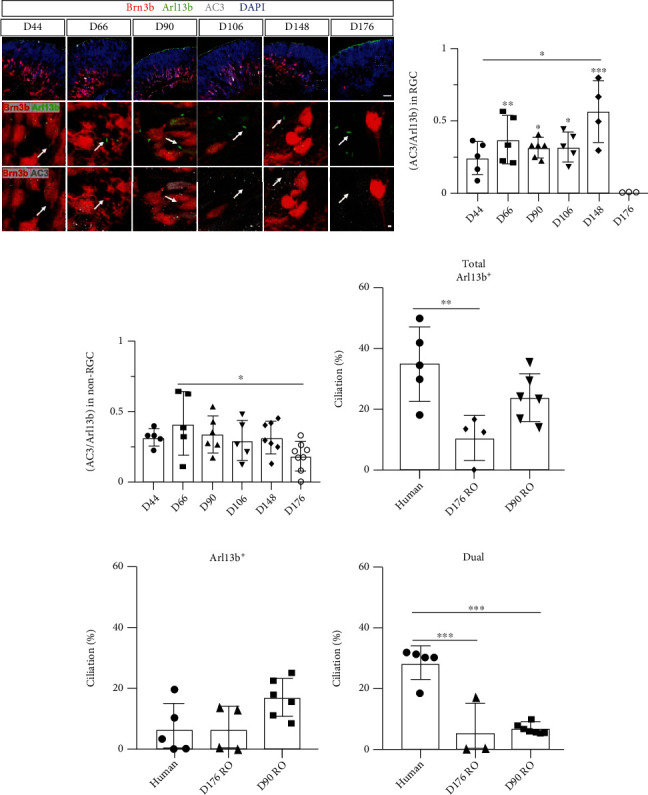
Decreased number of AC3-positive ciliated RGCs in retinal organoids. (a) Representative images illustrating retinal organoids immunostained with neuronal cilia marker AC3 (gray), and prototypical cilia marker Arl13b (green). RGCs are visualized by Brn3b-tdTomato (red), and nuclei are stained with DAPI (blue). Primary cilia labeled with AC3 and/or Arl13b can be localized to Brn3b-positive RGCs (white arrows). (b, c) Quantification of the ratio of AC3-positive cilia with respect to the total number of Arl13b-positive cilia in Brn3b-positive RGCs (b) and non-RGCs (c). (d-f) Quantification of precent ciliated RGCs in human retina, day 176 and day 90 of retinal organoids. Values represent mean ± SD.

Retinal organoid sections were imaged using a Zeiss LSM 880 scanning confocal microscope at 63× with 0.6× optical zoom (223 × 223*μ*m^2^). The images were taken every 0.3 *μ*m in the *z*-plane (20 slides per image) and maximum intensity projection images were further created from the Z-stacks. At least 5 images from each of 3-5 independent/individual retinal organoids were captured for further analysis. The number of cells and the overlap between them were calculated manually by one investigator using Zen software who was masked to the groups. In Figures 1 and 2, a cilium was counted when the Arl13b and Centrin3 signals were adjacent. Due to limited number of fluorescence channels, cilium was counted based on Arl13b signal in Figures 3, 4, and 5. In Figures 1(b) and 1(c), two rectangular boxes with a set width were drawn in each image, which determined that their length just covered the photoreceptor to ganglion cell layer of retinal organoids. Boxes were divided into outer and inner regions by the central dot. For Figure 1(b), the number of nuclei and primary cilia within the boxes was counted to determine the ciliation. In the remaining figures, cells and primary cilia were counted throughout the entire captured image.

### 2.6. Statistical Analysis

All statistical analyses were conducted using Graphpad8 (Prism) software. The results were presented as mean values ± standard deviation. For statistical analysis, unpaired *t*-test or one-way ANOVA was used to compare various groups. It was considered statistically significant if the *P* value was less than 0.05. The ARVO Statement for the Use of Animals in Ophthalmic and Vision Research was followed throughout the research.

## 3. Results

### 3.1. Primary Cilia Are Expressed in the Inner Part of hESC-Derived Retinal Organoids; Their Number Decreases in Later Developmental Stages

To investigate the spatial and temporal pattern of primary cilia expression during hESC-derived retinal organoid development, the Brn3b-Tdtomato hiPSC line was used, which was initially described [53]. After successful culture and organoid formation, immunostaining was performed on cryopreserved sections of the retinal organoids collected from day 44 to day 266. We employed antibodies that specifically detect two ciliary protein markers, Arl13b and Centrin3: Arl13b identifies ciliary axonemes, Centrin3 identifies basal bodies [45, 55]. An Arl13b fluorescence signal that aligned with a Centrin3 signal in confocal Z-stack images was considered a primary cilium (Supplementary Figure 1. Secondary-only control for Centrin3). In the positive control, Arl13b-positive cilia from photoreceptors were stably expressed and aligned at the edge of retinal organoids in all developmental stages, indicating the success of organoid differentiation and immunostaining [56, 57]. Since little is known regarding cilia in nonphotoreceptor cells in retinal organoids [46, 52], we focused our analysis on the inner retina during organoid development. Consistent with our previous reports in mouse retinas [31, 45], many ciliated cells (excluding photoreceptors) appeared in the inner parts of retinal organoids at all developmental time points (Figure 1(a)). Next, we assessed the number of cells (excluding photoreceptors) with cilia (ciliation) at different retinal organoid developmental stages. Of the time points analyzed, the number of ciliated cells was greatest on day 90 (29.01 ± 12.02%, *n* = 4). Interestingly, ciliation in the retinal organoids was significantly decreased on day 196 and day 266 compared to day 90 (day 196: 5.80 ± 3.20%, *n* = 4, ^∗∗^*P* = 0.003; day 266: 7.78 ± 4.42%, *n* = 3, ^∗^*P* = 0.014, One-way ANOVA, Figure 1(b)). To further investigate the spatial distribution of these ciliated cells, we divided the retina organoids into outer and inner regions of equal size (details in Methods) and assessed the number of ciliated cells in each region. Significantly more ciliated cells were distributed in the inner portion of the retina at most developmental stages (unpaired *t*-test, Figure 1(c)), suggesting that amacrine cells and RGCs comprise most ciliated cells during retinal organoid development. In summary, primary cilia were present in the inner portion of retinal organoids; their number reached a peak on day 90 and significantly decreased at later time points.

### 3.2. Primary Cilia Are Present in Chx10-Positive, GFAP-Positive, and PKC*α*-Positive Retinal Cells

To confirm the pattern of ciliation, we examined the inner retinal ciliated cells in a human eye by staining cryosections of human fetal retina (week 22) for ciliary markers, Arl13b and IFT88. As in hESC-derived retinal organoids, ciliated cells were present in the inner layer of the human retina (Figure 3(a)). To further explore the primary cilia in various retinal cell types during retinal organoid development, canonical cell markers, particularly for retinal progenitor, bipolar, horizontal, and astrocyte cells, were costained with Arl13b on retinal organoid sections at different developmental stages. We used Chx10 as a standard marker for retinal progenitor cells at early developmental stage [49–51]. Some, but not all, Chx10-positive cells contained primary cilia throughout retinal organoid development (Figure 3(b)). Next, we employed the PKC*α* antibody to examine the cilia status of rod-bipolar cells [47, 54, 58]. At day 266, PKC*α*-positive cells were detected on retinal organoid sections; only 4% of PKC*α*-positive cells contained primary cilia (*n* = 3). Interestingly, we did not detect ciliated cells that expressed Prox 1, a known marker for horizontal cells [47, 54, 58], in retinal organoids at day 176. This result suggests that Prox1-positive horizontal cells in retinal organoids do not harbor primary cilia at least at this time, which is consistent with our previous mouse data [31]. Lastly, using a GFAP antibody to visualize astrocytes [48], we observed expression of primary cilia in GFAP-positive astrocytes in day 266 retinal organoids. The cilia profile was also analyzed in proliferating cells on day 74 retinal organoids. Interestingly, Arl13b-positive cilia were only detected in 7.23% of Ki67-positive cells (Supplementary Figure 2 a total of 166 Ki67-positive cells were counted). Taken together, these results identify primary cilia on multiple retinal cell types, including retinal progenitor, horizontal, rod-bipolar, and astrocyte cells, and show that only horizontal cells express no detectable cilia during retinal organoid development.

### 3.3. Primary Cilia Are Present and Stable in AP2*α*-Positive Amacrine Cells and Their Subtypes in hESC-Derived Retinal Organoids

We previously found that amacrine cells in mouse and primate retinas contain primary cilia [31]. Amacrine cells are localized to the inner part of retina, where ciliated cells in retinal organoids were observed to be most abundant. To investigate whether amacrine cells in retinal organoids express primary cilia, we used AP2*α*, a well-accepted marker for amacrine cells [49, 54]. AP2*α*-positive amacrine cells in retinal organoids demonstrated primary cilia at all developmental stages, from day 44 to day 176 (Figure 4(a)). Quantitative analysis showed that the frequency (ciliation) and length of primary cilia displayed no significant differences in AP2*α*-positive amacrine cells among all developmental stages, suggesting that cilia are stably expressed in amacrine cells of retinal organoids (*n* = 3 or 4 per timepoint; ciliation: *P* = 0.561; length: *P* = 0.109, One-way ANOVA, Figures 4(b)–4(c)). To further explore the cilia profile in different subtypes of amacrine cells, we applied immunostaining for GAD67 (glutamate decarboxylase 67), a protein expressed in horizontal and amacrine cells during retinal development [59, 60]. Coimmunostaining for GAD67 and AP2*α* antibodies identified GAD67-positive amacrine cells; their cilia profile was assessed by Arl13b (Figure 4D). At day 44, only approximately 4.17 ± 6.25% of GAD67-positive amacrine cells expressed primary cilia (*n* = 3). This ciliated population was significantly increased at day 90 and day 148 (day 90: 30.80 ± 14.17%, *n* = 5, ^∗^*P* = 0.033; day 148: 31.22 ± 14.23%, *n* = 5, ∗*P* = 0.043, unpaired *t*-test, Figure 4(e)) indicating that cilia in GAD67-positive amacrine cells may participate in the development or differentiation of this subtype. Additional amacrine cell subtypes are positive for calretinin, which is expressed in aII amacrine cells and some RGCs [61–63]. Coimmunostaining retinal organoid sections for calretinin, AP2*α*, and Arl13b antibodies revealed a decreasing trend of ciliation in calretinin-positive amacrine cells during organoid development (*P* = 0.382, One-way ANOVA; Figures 4(f) and 4(g)). In summary, AP2*α* -positive amacrine cells and their subtypes in retinal organoids demonstrated stable expression of primary cilia, suggesting that retinal organoids are a feasible model to investigate the role of primary cilia in amacrine cell development and function.

### 3.4. Brn3b-Positive RGCs in Retinal Organoids Lose Primary Cilia at Later Development Stages

We and others have previously reported the presence of primary cilia within RGCs and their subtypes in mice and zebrafish [25, 45]. In this study, our efforts focused on characterizing the frequency and length of primary cilia during the development and differentiation of Brn3b-positive RGCs in retinal organoids. Coimmunostaining by Arl13b and Centrin3 identified primary cilia on Brn3b-tdTomato positive cells, indicating that the retinal organoid-derived RGCs contained primary cilia (Figure 2A). The number of Brn3b-positive RGCs was dramatically decreased in later stages and was barely detectable on day 176, which was consistent with other studies [49, 50, 52, 54]. Therefore, we only examined primary cilia between day 44 and day 176. Ciliated Brn3b-positive RGCs were most abundant on day 44; their ciliation was decreased at all later developmental timepoints. Quantitative analysis revealed significant decreases in ciliation at days 148 and 176 compared to day 44 (day 44: 43.57 ± 8.44%, *n* = 3; day 148: 19.46 ± 8.54%, *n* = 3; day 176: 19.76 ± 11.81%, *n* = 6; day 44 vs. day 148: ^∗^*P* = 0.044, day 44 vs. day 176: ^∗^*P* = 0.015, One-way ANOVA; Figure 2(b)). However, we observed no significant differences in ciliary length between days 44 to 176 of retinal organoid development (*P* = 0.700, One-way ANOVA; Figure 2(c)). Additionally, we examined the cilia profile in apoptotic RGCs on day 145 retinal organoids (*n* = 3) using caspase 3 (a reliable marker for apoptosis) and Arl13b antibodies. Interestingly, we found that majority of apoptotic RGCs (caspase3-positive and Brn3b-positive) were nonciliated, with only 12.04 ± 12.53% of cells were ciliated (Supplementary Figure 3). Many different subtypes of RGCs have been identified in the mammalian retina, a third of which express the calcium-binding protein, calretinin [45, 64]. We further assessed primary cilia in calretinin-positive; Brn3b-positive RGCs during retinal organoid development. In calretinin-positive; Brn3b-positive RGCs, ciliation on days 148 and 176 was significantly decreased when compared to day 44 (day 44: 47.08 ± 17.66%, *n* = 5; day 148: 7.29 ± 8.59%, *n* = 4; day 176: 16.67 ± 23.57%, *n* = 6; day 44 vs. day 148: ^∗∗^*P* = 0.007, day 44 vs. day 176: ^∗^*P* = 0.019, One-way ANOVA; Figure 2(e)). The primary cilia length of calretinin-positive, Brn3b-positive RGCs did not vary significantly during retinal organoid development (*P* = 0.346, One-way ANOVA; Figure 2(f)). We also used Islet2 and osteopontin as markers to investigate additional subtypes of RGCs. No cilia were observed in Islet2-positive; Brn3b-positive and osteopontin-positive; Brn3b-positive RGCs on day 148 (Figure 2(g)). Taken together, these results show that the ciliation and number of Brn3b-positive RGCs are significantly decreased in the later development stages.

### 3.5. Most Ciliated RGCs of Human Retina Are AC3-Positive

**Figure 6:**
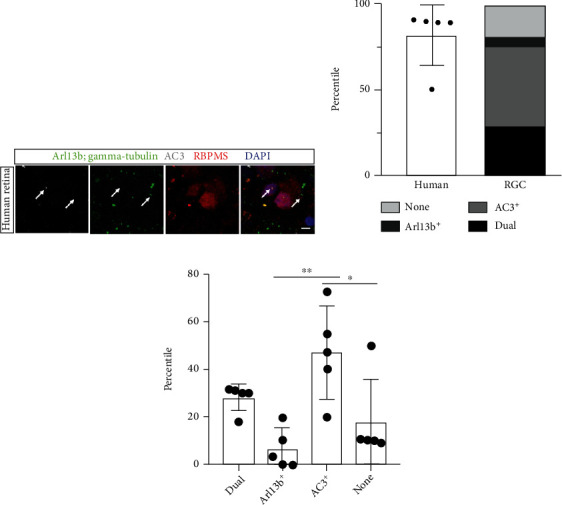
Ciliated AC3-positive RGCs are identified in human retina. (a) Representative images illustrating 68-year-old human retina immunostained with the neuronal ciliary marker, AC3 (gray), Arl13b and gamma-tubulin cilia marker (both in green). RGCs are marked by RBPMS (red), and nuclei are stained with DAPI (blue). White arrows indicate primary cilia. (b) Quantification of the population percentages of 4 subtypes of RGCs defined by ciliary markers. (c) Quantification of the populations of 4 subtypes of RGCs shows that AC3-positive ciliated RGCs are the most abundant (One-way ANOVA). Scale bar: 10 *μ*m; magnified, 1 *μ*m.

AC3, which mediates cAMP signaling that is essential for neuronal survival, has been found to be highly expressed on the primary cilia membrane of adult mouse RGCs [33, 42, 43, 45]. However, whether AC3-positive primary cilia are expressed in adult human RGCs remains unexplored. In the present investigation, we immunostained a healthy retina obtained from a 68-year-old patient with the RGC cell marker, RBPMS, and ciliary markers (AC3, Arl13b and gamma-tubulin) [65]. We first assessed the total ciliation of human RGCs, including both Arl13b- and AC3-positive primary cilia, in the peripheral area of retinal flatmounts (Figures 6(a) and 6(b)). Quantitative analysis showed that 82.0 ± 17.9% of human RGCs contained primary cilia (*n* = 89 total RBPMS^+^ cells counted). A group of human RGCs had no detectable primary cilia (neither Arl13b- nor AC3-positive), which was consistent with our rodent data [45]. Human RGCs were further divided into four subgroups based on the profile of their immunostaining for ciliary markers: cilia positive for Arl13b alone, AC alone, both Arl13b and AC, or neither. Interestingly, the Arl13b-positive primary cilia only accounted for 6.8% ± 8.5% of ciliated RGCs, whereas AC3-positive cilia accounted for significantly more, 47.1 ± 19.4%; 28.2 ± 5.6% expressed both markers; and 18.0 ± 17.9% expressed none (Arl13b-positive vs. AC3-positive: ^∗∗^*P* = 0.002, AC3-positive vs. none: ∗*P* = 0.024, One-way ANOVA; Figure 6(c)). Taken together, our findings demonstrate that most ciliated human RGCs express AC3-positive primary cilia, suggesting a key role in adult RGC homeostasis.

### 3.6. Decreased AC3-Positive Ciliated RGCs in Retinal Organoids

Because AC3-mediated cAMP signaling is crucial for neuronal survival and our previous data demonstrate the presence of AC3-positive primary cilia in human and mouse adult RGCs *in vivo*, we further examined AC3 expression in the cilia of RGCs in retinal organoids. AC3 and Arl13b were costained on retinal organoid sections at different developmental stages (Figure 5(a)). We were unable to identify solely AC3-positive primary cilia on retinal organoid sections due to strong background staining and weak fluorescence signal of the AC3 channel. We evaluated the population of AC3-positive primary cilia in Brn3b-positive RGCs by assessing the ratio of AC3/total Arl13b. Based on previous human RGCs data, this total Arl13b group included groups that were positive for Arl13 alone and for both Arl13 and AC3. Quantitative analysis displayed a trend toward an increase of the AC3/total Arl13b ratio, with a peak at day 148 followed by a significant decrease at day 176, which indirectly reflected the number of AC3-positive primary cilia. Specifically, compared to day 44, the ratio of AC3/total Arl13b in Brn3b-positive RGCs was significantly greater on day 148. However, at day 176, there were no detectable AC3-positive primary cilia in the Brn3b-positive RGCs (day 44: 24.21 ± 11.60%, *n* = 5; day 148: 56.67 ± 21.60%, *n* = 4; day 176 : 0 ± 0%, *n* = 3; day 44 vs. day 148: ^∗^*P* = 0.013, day 44 vs. day 176: ^∗^*P* = 0.019, day 66 vs. day 176: ^∗∗^*P* = 0.008, day 90 vs. day 176: ^∗^*P* = 0.025, day 106 vs. day 176: ^∗^*P* = 0.026, day 148 vs day 176: ^∗∗∗^*P* < 0.001, One-way ANOVA; Figure 5(b)). The variation in the quantitative analysis was large on day 148 and day 176 due to the limited number of RGCs remaining in the retinal organoids. Additionally, we examined the AC3/total Arl13b ratio in non-RGC retinal cells (except cilia in photoreceptors) and again found a significant decrease in the ratio at day 176 compared to day 66 (^∗^*P* = 0.047, One-way ANOVA, Figure 5(c)). However, at day 66, when the ratio was at its lowest in non-RGC retinal cells, 18.57 ± 10.44% of cilia were still AC3-positive. We also used human RGC data to compare RGCs in the total Arl13b group, Arl13b-positive, and dual marker group to the data for day 90 and 176 in retinal organoids (Figures 5(d)–5(f)). In the total Arl13b group, ciliation was significantly greater in human RGCs than in day 176 organoids, suggesting that the total Arl13b-positive cilia number was decreased in organoid RGCs compared to human RGCs (human vs. day 176: ^∗∗^*P* = 0.006, One-way ANOVA; Figure 5(d)). Arl13b-positive cells did not differ significantly among the three groups, human RGCs and retinal organoids at day 90 and 176 (Figure 5(e)). Interestingly, for the dual marker group, the ciliation was significantly higher in human RGCs than in retinal organoids at both days 90 and 176, indicating that RGCs expressing AC3-positive cilia were far fewer in retinal organoids than in human RGCs at the later stages, or completely lost (human vs. day 90: ^∗∗∗^*P* < 0.001, human vs. day 176: ^∗∗∗^*P* < 0.001, One-way ANOVA; Figure 5(f). Taken together, assessment of AC3 expression in the cilia of retinal organoid derived RGCs revealed dramatically decreased expression in the later developmental stages, which was the opposite pattern of that observed in human RGCs, suggesting that the loss of AC3 expression and associated signaling pathways in primary cilia contribute to RGC depletion in retinal organoids at later stages.

## 4. Discussion

In this study, we examined the distribution of primary cilia in hESC-derived retinal organoids throughout development, with a focus on amacrine cells and RGCs. First, we observed a group of ciliated cells located in the inner portion of the retinal organoids that significantly decreased at later developmental stages. We then found that ciliated Brn3b-positive RGCs were also dramatically decreased at later time points, whereas primary cilia were stably expressed in AP2*α*-positive amacrine cells at all developmental stages. Additionally, we observed that expression of AC3, a key ciliary protein, was significantly decreased in the primary cilia of RGCs at late developmental stages, compared not only to earlier developmental stages but also to adult human RGCs. This result suggests that the decrease of AC3 expression in primary cilia and its effects on related downstream signaling pathways contribute to RGC loss in later stages of retinal organoid development, a hypothesis that can be tested in future work.

The role of primary cilia in amacrine cells, though expressed in mouse and primate retinas [31, 66], remains unknown [28, 31, 66, 67]. Genetic knockout of *Ift88*, which inhibits primary cilia assembly, from vGluT3 amacrine cells showed limited impact on the visual pathway in mice [31]. In this study, we detected ciliated AP2*α*-positive amacrine cells in the retinal organoids, which is consistent with previous findings [28, 31]. Surprisingly, the ciliation remained stable at all development stages that we examined, indicating that the retinal organoid is a powerful tool for future studies dissecting the role of primary cilia and their regulated signaling pathways in amacrine cell development and differentiation. Our data on amacrine subtypes suggest that primary cilia serve various roles in different amacrine subtypes. For instance, we observed very few ciliated GAD67 amacrine cells in the early developmental stages, followed by a significant increase in ciliation in the later stages. We also observed a different pattern of ciliary expression in amacrine cells containing calretinin: despite an overall trend toward slight decrease, their ciliation displayed no significant differences at different developmental stages. This data prompted us to hypothesize that primary cilia play an important role only in later developmental stages for GAD67 amacrine cells, but in both early and late stages for calretinin amacrine cells. Further studies could focus fruitfully on cilia-related signaling pathways, such as Sonic hedgehog and Wnt, to identify molecular mechanisms contributing to the development and differentiation of amacrine cells and their differences in diverse subtypes.

The gradual progressive loss of RGCs that occurs in stem-cell derived retinal organoids in later developmental stages has presented an unmet challenge to using them as an ex vivo model for glaucoma and drug screening [48, 50, 52, 53, 68]. Primary cilia are known to be essential for vertebrate nervous systems development by regulating neuronal patterning, proliferation, and migration, and cilia-related dysfunction leads to ocular disorders, including retinal degeneration, retinitis pigmentosa, rod-cone dystrophies, and optic neuropathies [3, 55, 69–72]. In this study, we found that the decrease in number of ciliated RGCs in the organoids over time was greater than the decrease in the number of surviving RGCs. However, it is known that RGCs are typically ciliated, except for a subset of alpha-RGCs, in the adult human and mouse [45, 66]. Several mechanisms could account for our observation of fewer ciliated RGCs than expected are as follows: (1) disassembly of primary cilia is a component of apoptosis during RGC degeneration (Supplementary Figure 3); (2) defective primary cilia and related signaling pathways contribute to RGC loss; and (3) disassembly of primary cilia disrupts RGC differentiation, which enhances RGC degeneration. Further studies will be required to determine the mechanism responsible. The limitations of the present study include the very small sample number of adult human retinas, which serve as a gold standard for comparison to the hESC-derived organoids. However, our findings receive support from several earlier reports that most RGCs are ciliated in mouse, human, and cat retinas [45, 66], suggesting that primary cilia are present in the majority of adult RGCs in most mammalian species.

Interestingly, many of the ciliated RGCs in retinal organoids contained only one of the prototypical cilia markers, Arl13b. This is different from observations we have made in both mouse and human RGCs in which most of the cilia are positive for AC3 [45]. One implication of this difference is that, since the organoid RGCs lack AC3, many of them do not have primary cilia at all and therefore do not survive in the organoids because AC3 plays an essential role in RGC homeostasis. AC3 catalyzes the conversion of ATP to cAMP, which is an important process for several signaling pathways, including calcium and G-protein coupled receptor signaling, both of which play important roles in neuronal function [42–44, 73–77]. Absence of AC3 expression also resulted in abnormal axon projections from olfactory sensory neurons in mouse [44], implying that loss of AC3 expression may also disrupt axon projections in organoid RGCs. Additional studies will be required to determine how primary cilia and their proteins contribute to both RGC axon projection and RGC loss and survival. The results of these studies may help to extend the survival time of cultured RGCs in stem-cell derived retinal organoids, which would increase their value in disease modeling and drug screening.

In summary, this study analyzed the presence of cilia in many of the cell types that develop in retinal organoids with a focus on inner retinal neurons, which provide a valuable platform for analyzing retinal development and for modeling human diseases (RGC degeneration). We observed that most cells are ciliated throughout development, although the number of ciliated cells is reduced in later stages of development. Further research is required to better understand the interrelationships between primary cilia, AC3, and RGCs that contribute to retinal cell survival and function.

## Data Availability

This article contains the data used to support the results of this study.

## Supplementary Materials

Supplementary Materials Supplemental Figure 1:secondary-only control images for Centrin3 staining. Representative images showing secondary antibody (Alexa Fluro 647, in white) with DAPI staining (in blue) on day 145 retinal organoid sections. Scale bar: 10 *μ*m; magnified, 5 *μ*m. Supplementary Figure 2: few Ki67-positive cells are ciliated in day 74 retinal organoids. Representative images illustrating Ki67-positive cells (in red) and Arl13b-positive cilia (in green) on day 74 retinal organoids (*n* = 2). Arrows pointed to primary cilia. DAPI stain nuclei in blue. Scale bar: 10 *μ*m; magnified, 5 *μ*m. Supplementary Figure 3: few caspase 3-positive; Brn3b-positive cells are ciliated in day 145 retinal organoids. Representative images showing the caspase 3-positive; Brn3b-positive RGCs on day 145 retinal organoids (*n* = 3). Caspase-3 signal showed in gray, Brn3b signal showed in red, Arl13b signal showed in green, and DAPI signal showed in blue. Arrows pointed to an example cell. Scale bar: 10 *μ*m; magnified, 5 *μ*m.
