# Supplementary figures and images for: Characterization of Primary Cilia Formation in Human ESC-Derived Retinal Organoids
Source: Stem Cells Int. 2023 Jan 13;2023:6494486. doi: 10.1155/2023/6494486 (PMC9859708; doi:10.1155/2023/6494486)

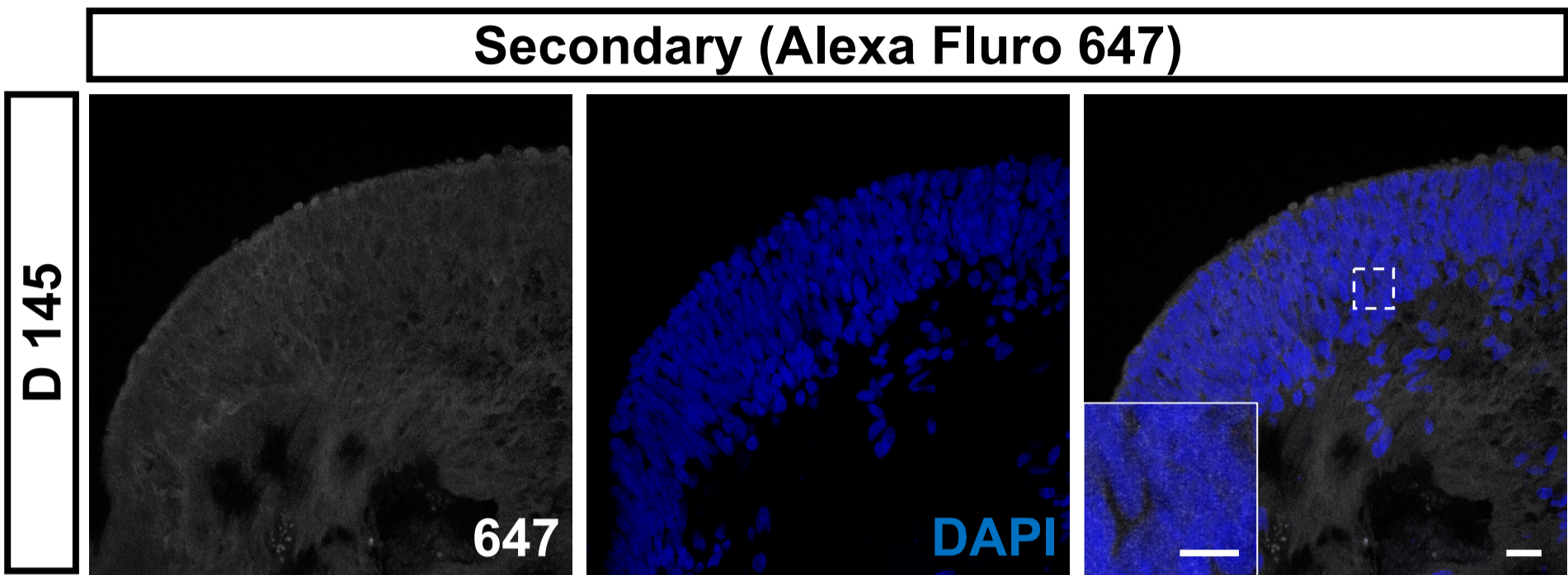

Supplementary figure 1.

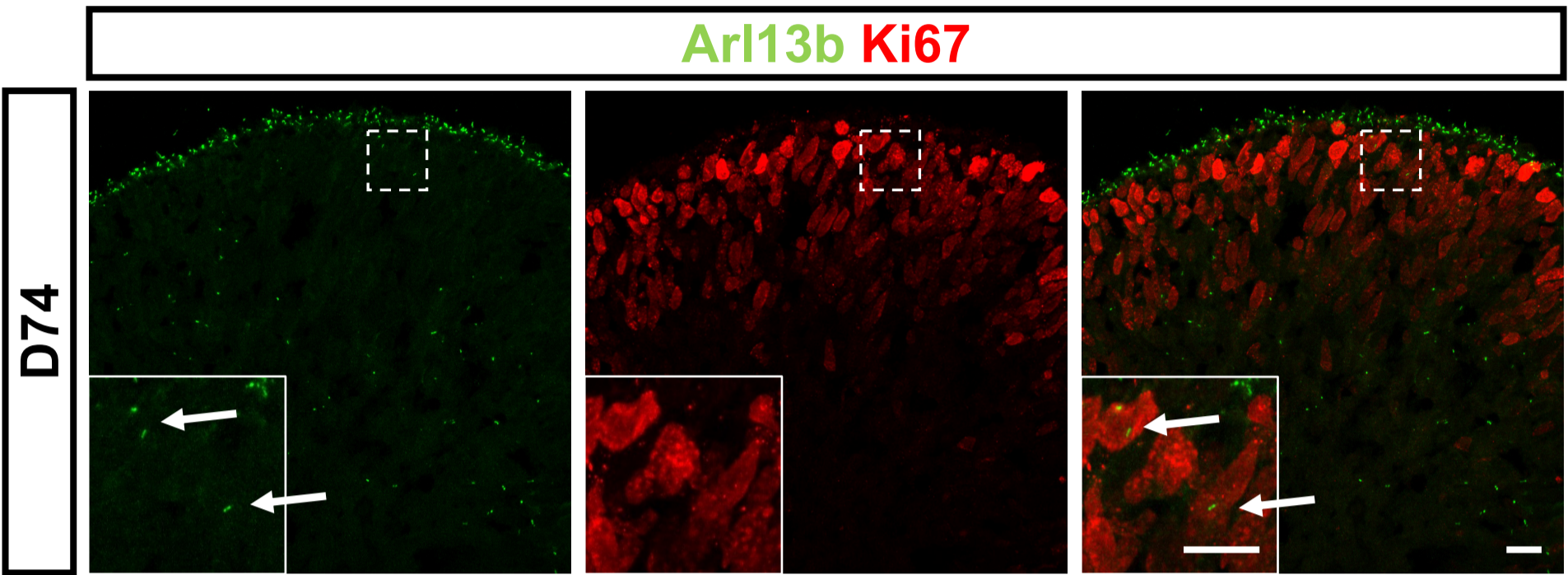

Supplementary figure 2.

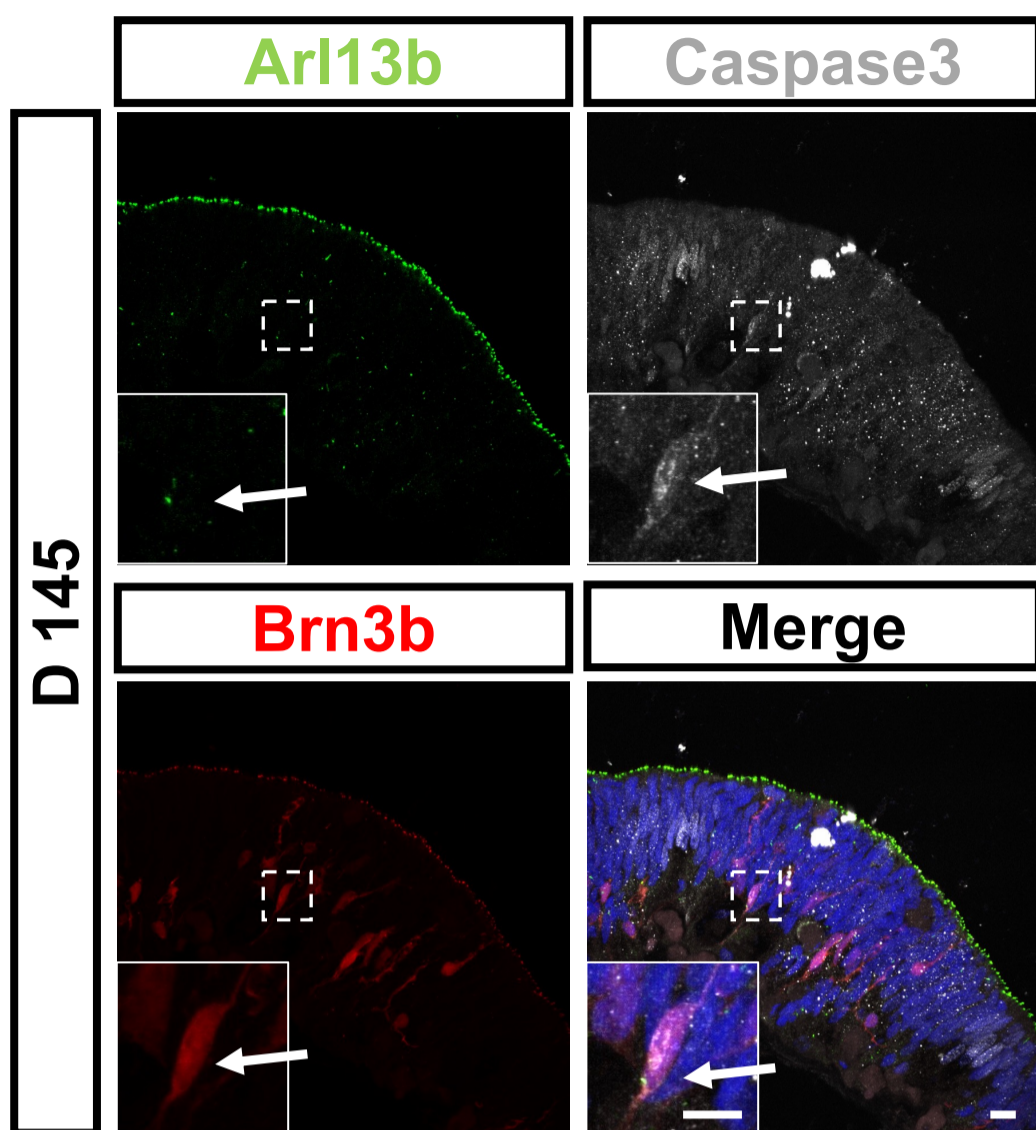

Supplementary figure 3.

Supplement: Supplementary Materials — Supplemental Figure 1:secondary-only control images for Centrin3 staining. Representative images showing secondary antibody (Alexa Fluro 647, in white) with DAPI staining (in blue) on day 145 retinal organoid sections. Scale bar: 10 μm; magnified, 5 μm. Supplementary Figure 2: few Ki67-positive cells are ciliated in day 74 retinal organoids. Representative images illustrating Ki67-positive cells (in red) and Arl13b-positive cilia (in green) on day 74 retinal organoids (n = 2). Arrows pointed to primary cilia. DAPI stain nuclei in blue. Scale bar: 10 μm; magnified, 5 μm. Supplementary Figure 3: few caspase 3-positive; Brn3b-positive cells are ciliated in day 145 retinal organoids. Representative images showing the caspase 3-positive; Brn3b-positive RGCs on day 145 retinal organoids (n = 3). Caspase-3 signal showed in gray, Brn3b signal showed in red, Arl13b signal showed in green, and DAPI signal showed in blue. Arrows pointed to an example cell. Scale bar: 10 μm; magnified, 5 μm. [file 6494486.f1.pdf]
